# In small animal surgery are alcoholic hand rubs superior to scrubbing brushes and antimicrobial soap at reducing bacterial counts?

**DOI:** 10.18849/ve.v10i4.51

**Published:** 2025-11-12

**Authors:** Alison Mann+

**Keywords:** ALCOHOL HAND RUB, ANTIMICROBIAL SOAP, BACTERIAL COUNT, SCRUB BRUSH, SMALL ANIMAL, SURGERY

## Abstract

**Knowledge Summary Update:**

This paper is an update to 'In Small Animal Surgery Are Alcoholic Hand Rubs Superior to Scrubbing Brushes and Antimicrobial Soap at Reducing Bacterial Counts?' by Mann (2016).

Please click the link to view the original paper: https://doi.org/10.18849/ve.v1i4.51 .

**PICO question:**

In small animal surgery are alcoholic hand rubs superior to scrubbing brushes and antimicrobial soap at reducing bacterial counts?

**Clinical bottom line:**

**Category of research:**

Prevalence.

**Number and type of study designs reviewed:**

Two studies. One prospective randomised controlled trial and one clinical trial.

**Strength of evidence:**

Moderate.

**Outcomes reported:**

That alcohol hand rubs are as effective, if not more, than antimicrobial soap for presurgical hand preparation when used according to the manufacturer guidelines.

**Conclusion:**

Alcohol hand rubs are as effective than antimicrobial soaps at presurgical hand preparation. If human healthcare studies were included in this Knowledge Summary, the results would likely be stronger and more conclusive so this should be considered for a separate knowledges summary. There are also added benefits to using alcohol hand rubs such as saving water and quicker preparation of the surgical scrub team.

## How to apply this evidence in practice

The application of evidence into practice should take into account multiple factors, not limited to: individual clinical expertise, patient’s circumstances and owners’ values, country, location or clinic where you work, the individual case in front of you, the availability of therapies and resources.

Knowledge Summaries are a resource to help reinforce or inform decision-making. They do not override the responsibility or judgement of the practitioner to do what is best for the animal in their care.

## Clinical scenario

You have been asked to look up the current literature on whether alcohol hand rubs perform better than antimicrobial soap and scrub brushes with a view to changing your protocol on presurgical hand preparation.

## The evidence

The majority of the literature found was author narrative or looking at the current attitudes and habits that veterinary surgeons have regarding surgical hand preparation. Two relevant papers were found comparing alcohol hand rubs (AHRs) to traditional methods of hand preparation (Chou et al., 2016; Verwilghen et al., 2011). They differed slightly in that the first paper (Chou et al., 2016) did not use an abrasive brush in any of the regimes; it only looked at alcohol in different formulations against antimicrobial soap. The second paper (Verwhilgen et al., 2011) did look at the traditional hand scrub with a brush and antimicrobial soap as one of the comparisons.

Many human studies were found, with a number of them having relevance in all areas of the PICO question apart from the population, so these were excluded.

## Summary of the evidence

**Chou et al. (2016):** Antibacterial Efficacy of Several Surgical Hand Preparation Products Used by Veterinary Students **Aim: **To compare the antibacterial efficacy of different surgical hand antisepsis protocols used by veterinary students.

Population:	Third year veterinary students (University of Prince Edward Island, Canada).
Sample size:	45 students.
Intervention details:	The participants were randomly assigned to carry out 4 of the 12 possible combinations of presurgical hand preparation: Non-abrasive hand scrub with 4% Chlorhexidine gluconate (CHG) antimicrobial soap. Alcohol hand rub (AHR) with 30% 1-propanol and 45% 2 –propanol. AHR with 70% 2 –propanol. AHR with 61% ethanol solution with 1% CHG. All the above products could have had any one of the below contact times thus making 12 possible combinations from 1.5, 3, or 5 minutes. Using shuffled cards, the participants were assigned 4 of the possible 12 combinations.
Study design:	Prospective randomised controlled trial.
Outcome Studied:	To compare the antibacterial efficacy of different surgical hand antisepsis protocols used by veterinary students both at the recommended and at extended contact times. Antibacterial efficacy was assessed before surgical hand preparation, after surgical hand preparation and at the end of surgery. Reductions in bacterial colony forming units and positive aerobic culture rates were compared using multivariable analysis or variance and multivariable logistic regression.
Main Findings (relevant to PICO question):	After hand preparation the AHR with 61% ethanol/1% CHG and the CHG non abrasive antimicrobial soap scrubs were more effective at reducing log colony forming units (CFUs) when used at the manufacturer recommended contact time. Increasing contact time for any of the products did not have an effect on bacterial reduction immediately after hand preparation. At the end of surgery, the product used, the contact time and the product/ contact time interaction all had effects on CFU reductions. At the end of surgery, the AHR with 61% ethanol/1% CHG and the AHR with 30% 1-propanol and 45% 2- propanol had significantly increased bacterial reductions with increasing contact time. At the end of surgery, at the manufacturer recommended contact time the AHR with 61% ethanol/1% CHG had significantly higher CFU reduction compared to the AHR with 70% 2-propanol.
Limitations:	Authors and participants not blinded to the products used or the contact time. Veterinary students may not be as experienced in the methods of presurgical hand preparation. There were guidelines, however there was likely to be some variability between participants. There was some variability between surgical times (57–255 mins) which may have affected the final samples taken. However, in the discussion it is stated that the average surgery time was relatively short. There was no comparison with an abrasive scrubbing brush; the scrubbing method used a sponge as opposed to a brush.

**Verwilghen et al. (2011):** Surgical hand antisepsis in veterinary practice: Evaluation of soap scrubs and alcohol based rub techniques **Aim: **To compare the efficiency of medicated soaps and a hydro-alcoholic solution prior to surgery using an in-use testing method in a veterinary setting.

Population:	Small animal and equine surgeons.
Sample size:	3 equine surgeons and 2 small animal surgeons. A total of 64 samples were obtained for AHRs (Sterilium) (50 equine, 14 small animal) and 30 obtained for antimicrobial soap (CHG) (20 equine, 10 small animal).
Intervention details:	A preliminary study was carried out comparing Povidone Iodine (PVP), CHG and Sterilium. Following this preliminary study it was found that the actions of the PVP were not comparable to the others and so the clinical in use study was carried out only using CHG and Sterilium. Sample sizes are discussed above. The CHG was used in a 5 minute scrubbing technique and the Sterilium was used according to manufacturer’s instructions at 1.5 minutes after a 1 minute’s hand wash with neutral soap. Fingertips were pressed for 10 seconds onto a blood agar plate (separate for each hand) and bacterial growth was quantified by counting the CFUs grown after 24 hours of incubation. This was performed prior to hand antisepsis, immediately after hand antisepsis and after surgery during which the surgeon was double gloved (the first pair discarded after draping the patient). Mean surgery time was 1.5 hours.
Study design:	Clinical trial.
Outcome Studied:	To compare AHRs to CHG in a surgical setting.
Main Findings (relevant to PICO question):	A preliminary study was carried out comparing PVP, CHG, and Sterilium and found that PVP was not comparable to the other 2 products and so this was not taken forward into the further study. 4 Sterilium samples were excluded due to contamination during surgery, 1 was excluded due to an infected wound by the nail of one of the surgeons creating an extreme growth of staphylococcus aureus. Prior to hand antisepsis samples were significantly different to after hand antisepsis and after surgery samples for both products. No difference was found in CFUs between after hand antisepsis and after surgery samples for Sterilium. The clinical in use trial found that there was a significantly greater reduction factor for the Sterilium compared to CHG.
Limitations:	Residual activity of CHG is difficult to assess without the use of a neutralising agent as bacteriostatic concentrations of the CHG will remain. The decision was made to not use a neutralising agent as it was not used in similar studies. More than double the amount of samples were gained for the Sterilium group, although some of these did have to be discarded.

## Appraisal, application and reflection

Two relevant studies were found for this particular PICO question (Chou et al., 2016; Verwilghen et al., 2011), which both conclude that alcohol is as effective, if not more, than antimicrobial soap for presurgical hand preparation when used according to the manufacturer guidelines. The alcohol hand rubs (AHRs) used in the two studies, however, were of different formulations. In the first study (Chou et al., 2016) there were 2 formulations of alcohol used: propanol (of different strengths) and alcohol with 1% Chlorhexidine gluconate (CHG), of which the alcohol with CHG was found to be the most effective. In the second study (Verwilghen et al., 2011) the only alcohol formulation used was propanol (Sterilium). The PICO question only specifies AHRs rather than particular formulations so both studies are relevant. The results of this study (Verwilghen et al., 2011) agreed with previous studies studies carried out in this area (Parienti et al., 2002; Kampf & Osteomeyer, 2005; Löffler & Kampf, 2008; Tanner et al., 2008).

Another difference between the studies was that Chou et al. (2016) did not use an abrasive method of hand scrubbing as one of the comparisons; their non-abrasive scrub method was with the use of a sponge rather than bristles. As the PICO question in this instance was asking for a comparison between scrubbing brushes and AHRs there are some discrepancies between this and the PICO question, but the decision was made to include it. Verwilghen et al. (2011) did use the more traditional surgical scrub with a brush as one of their variables, making it very suited to the PICO question.

On reflection, whether in human or veterinary surgery, the end point of presurgical hand antisepsis is the same; to have reduced bacterial colony forming units on the hands. The author therefore feels that a future Knowledge Summary with a slight change to the PICO to include human surgery would still be relevant to veterinary professionals to draw evidence from.

## Methodology

**Search Strategy:** 

Databases searched and dates covered:	CAB Abstracts via the OVID interface: 1973 to 2024 Week 04 PubMed via the NCBI interface: 1973 to 2024 Week 04
Search strategy:	CAB Abstracts: Small animal or veterinary surgery or companion animal or (cats or cats or feline or felis or dogs or dog or canis or canine) or small animal surgery and (chlorhexidine gluconate or povidone iodine or antimicrobial soap) or scrubbing brush or (hand and scrub) or scrub and (alcohol rub or alcoholic rub or ethanol or propanol) or (hand and rub) PubMed: Small animal or veterinary surgery or companion animal or (cats or cats or feline or felis or dogs or dog or canis or canine) or small animal surgery and (chlorhexidine gluconate or povidone iodine or antimicrobial soap) or scrubbing brush or (hand and scrub) or scrub and (alcohol rub or alcoholic rub or ethanol or propanol) or (hand and rub)
Dates searches performed:	9 Feb 2024

**Exclusion / Inclusion Criteria:** 

Exclusion:	Large/farm animal studies, human studies, narrative reviews, any studies on skin preparation of patients as opposed to the surgeon, any studies that are not relevant to the PICO question.
Inclusion:	Small animal/companion animal studies comparing the 2 hand preparation techniques.

**Search Outcome:** 

Database	Number of results	Excluded – narrative	Excluded – not relevant to the PICO	Excluded – human study	Excluded – large animal	Total relevant papers
CAB Abstracts	33	0	32	0	0	1
PubMed	1047	1	1035	8	2	1
Total relevant papers when duplicates removed						2

## ORCiD

Alison Mann: https://orcid.org/0000-0002-5785-2883

## Conflict of Interest

The author declares no conflicts of interest.
